# The Use of Structural Templates in Protein Backbone Modeling

**DOI:** 10.6028/jres.094.009

**Published:** 1989

**Authors:** Lorne S. Reid

**Affiliations:** Allelix Biopharmaceuticals 6850 Goreway Drive Mississauga, Ontario Canada, L4V 1P1

**Keywords:** *α* helix, *β* turn, compact domains, modeling, protein structure, sequence profiles, structure prediction, templates

## Abstract

The procedures used to model a protein structure are well established when the novel protein has high sequence similarity to a protein of known structure. Many proteins of interest have low (i.e. <50%) sequence similarity to any known structure. In these cases new approaches to prediction of structure are required.

The use of sequence profiles which relate sequence to known structure has been proposed as one method to assign local regions of structure. As a first stage, templates or “icons” of the many relevant substructural motifs found in proteins must be defined. The sequences which gave rise to these structures are then aligned and a weighted profile obtained.

Average structures of the 8 and 12 residue helix-turn and turn-helix motifs have been prepared. These coordinate templates were then used to scan through the Brookhaven protein structural database for similar, superimposable fragments. A composite template of 100 similar fragments for each element was found to be internally consistent to a rmsd=0.92 Å for HT8, 1.54 Å for HT12, 0.41 Å for TH8 and 1.40 Å for TH12. All of the sequences, from these structures, were then used to create an overall sequence profile.

The four sequence profiles were scanned against the amino acid sequences of the proteins in the Brookhaven database: tertiary structure was correctly identified only about 10% of the time. This value is too low for predictive purposes. However, it could be increased by checking for multiple occurrences of the template in one protein.

## 1. Introduction

The process of protein modeling relies upon the database of structures determined principally by x-ray crystallography or, more recently, 2-D NMR techniques. As a first step in modeling, the degree of sequence similarity of a novel protein is compared to all proteins of known structure. Given high sequence similarity (>50%) the techniques of homology modeling will certainly be used [1–7]. The effectiveness of this process has been demonstrated in the construction of models of insulin-like growth factor [8], t-PA [9], and immunoglobulin variable domain [10] to name a few. However, many proteins of interest have a lower degree of homology or obvious insertions or deletions in their sequence. Any methods which can be used to predict the structure of these proteins are of great interest to experimentalists and theoreticians alike.

The secondary structure of a protein can be predicted with methods such as Chou-Fasman but only to some 65% accuracy [11,12]. To improve upon this, the use of sequence specific profiles has been proposed [1,13,14], The sequence specific requirements of *β* turns [15], N-cap, C-cap *α* helices [16] and proline-kinked *α* helices [17] have been previously defined. Also, the sequence requirements of large domains are known for the globin fold [18,19], and the immunoglobulin fold [20].

A major assumption in this procedure is that certain linear amino acid sequences give rise to specific structural elements [21–23]. Many different approaches have been taken to identify zones in proteins which are very closely packed [24–32], Most methods are computationally intensive; one simple method is to count the number of residues which he within a sphere of a given radius around any atom. To prepare a profile, the relevant fragments are extracted from all proteins of known structure and aligned in space. The amino acid types are then checked at each residue position and a weighted sequence profile determined. Any novel amino acid sequence can then be checked against a bank of such known profiles and the most likely tertiary fragments identified. This procedure differs from the standard predictive methods of secondary structure in that it attempts to assign specific three-dimensional structure on the basis of sequence and not just regions of secondary structure.

In this work, two examples of both turn-helix and helix-turn structures were chosen for study. These structures were previously identified by Zefus as highly compact structures which were repeated throughout many protein structures [31]. The purpose of this work is to outline some of the steps involved in the identification of relevant templates and their application to structure prediction.

## 2. Methodology

All programs were written in Fortran 77 and run on a VAX 11/750 under the VMS rev 4.7 operating system.

### 2.1 Preparation of Stage I Templates

The number and identity of residues which surround each residue in the protein lysozyme (Brookhaven code 1LZ1) were determined. The radius of the sphere checked around each atom was over the range of 3.0 to 8.0 Å.

### 2.2 Identification of Average Structural Template Coordinates

For the purposes of this work four structural units of a known compact nature were used. These were the 8 residue helix-turn (HT8), 12 residue helix-turn (HT12), 8 residue turn-helix (TH8), and 12 residue turn-helix (TH12) domains as assigned by Zefus [31].

#### 2.2.1 Preparation of Stage I Templates

The backbone coordinates of each member associated with a structural template were superimposed using a conjugate gradient rotation/translation function. The root mean square deviation (rmsd) of each member to every other member was calculated for both the main-chain and side-chain atomic positions.

If a particular member appeared to be significantly different from all the other members it was discarded from further consideration. The mean *X, Y, Z* coordinates of the main-chain atoms were calculated from the fragments under consideration. This coordinate set was identified as a stage I template.

#### 2.2.2 Preparation of Stage II Templates

Only proteins in the Brookhaven database (release October 1987) with a resolution of better than 2.5 Å were used in this work [33]: 82 non-homologous proteins, 177 proteins in total were used in this subset of the database. The 100 fragments with the lowest rmsd to the stage I template were rank ordered and the average coordinate set calculated. Finally, the average of the standard deviation of the errors in the *X, Y*, and Z coordinates was determined. This new coordinate set was identified as stage II template.

### 2.3 Amino Acid Sequence Profiles

The amino acid sequences used to prepare the stage II template were assembled with the programs of the University of Wisconsin Genetics Computer Group (Ver 5.2) [34]. A sequence profile was prepared with the program PROFILE [13]. The Protein Identification Resource/NBRF (PIR) (Rel 15.0) database [35] of amino acid sequences was scanned with the program PROFILESEARCH and alignments calculated with PROFILESEGMENTS. A subset of the PIR database, which corresponded to the proteins used in the Brookhaven database, was also checked for alignments to the calculated profiles.

## 3. Results

For the purposes of modeling or structure prediction it is necessary to clearly define substructural elements. A number of canonical structures such as *α* helices, *β* sheets or larger super-secondary elements such as Greek keys, or *α-β-α* units are well known. However, irregular or compound elements can have a very high packing density. Inter-residue contact plots are a convenient method for identification of both the contiguous and discontinuous zones of high density (data not shown).

**Figure 1:**
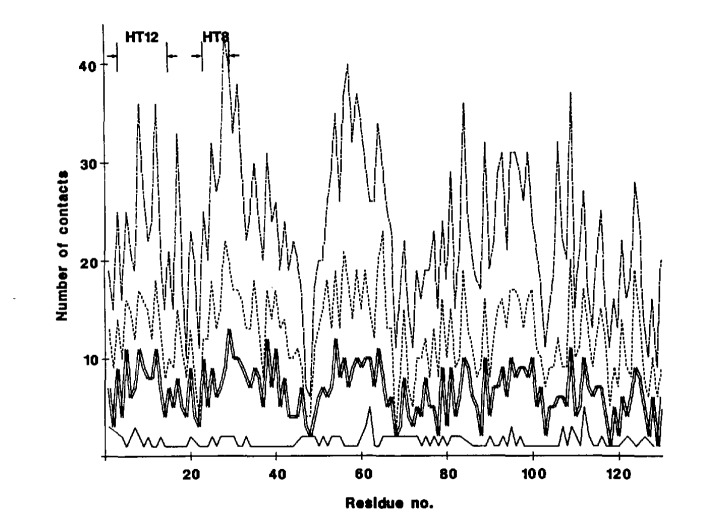
Nearest neighbor contacts in lysozyme (1LZ1) as a function of interatomic distance: 3.0 Å (–––), 4.0 Å (===), 6.0 Å (– – – –), 8.0 Å (–-–-). The TH12 and TH8 motifs exist in the protein at the identified regions of high packing density.

The number of contacts which a particular residue makes with its neighbors increases in a linear way with the size of the probe distance [36]. As shown in figure 1 for lysozyme (ILZl) beyond a shell size of 4.0 Å the shape of the compact domain did not change; there was an increase only in the number of residues involved. Two of the structural templates under investigation exist in the lysozyme structure and occur in regions of high packing density. Neither of the motifs in lysozyme were used to generate the stage I templates.

The fragments used for the preparation of stage I templates are given in table 1. A number of elements originally identified by Zefus as compact turn-helix 8 motifs were rejected for use in the preparation of the stage I TH8 template. Rejection was based upon an average rmsd, of the fragment to all other members of the test set (main-chain atoms only), of 1.5 Å greater than the average rmsd for all residues in the *N×N* test set.

Superimposition of the coordinate sets was based solely upon the backbone atoms. Those side-chain atoms which had equivalent atom names at superimposed residues were checked for structural homology. For example, if the backbones of alanine and cystine were superimposed the rmsd was determined for the C*β* atom position. On average, 1.5 side-chain atomic positions could be superimposed at each residue over all the paired coordinate sets.

The turn-helix 8 stage I template had the greatest degree of structural homology for both main-chain and the superimposable side-chain atoms. In each stage I template the greatest diversity occurred in the turn region: the helix was well defined. This may relate to actual differences in the structure and partly to the difficulty of building the original protein structure into x-ray density associated with irregular elements such as these turns. Alternatively, this may indicate that average rmsd error is a relatively insensitive indicator of similarity between protein fragments.

The Brookhaven protein database was scanned for the best 100 fragments which could be superimposed onto the stage I template. Due to the existence of multiple forms and multiple chains in a protein the database has significant redundancy. However, these redundant fragments had minor variations in three dimensional structure. Keeping and averaging these redundant forms reduced the structural error associated with the motif as found in any one particular crystal structure. Table 2 indicates the average rmsd values of the top 50 and top 100 fragments which were found in this manner for each template type.

**Figure 2:**
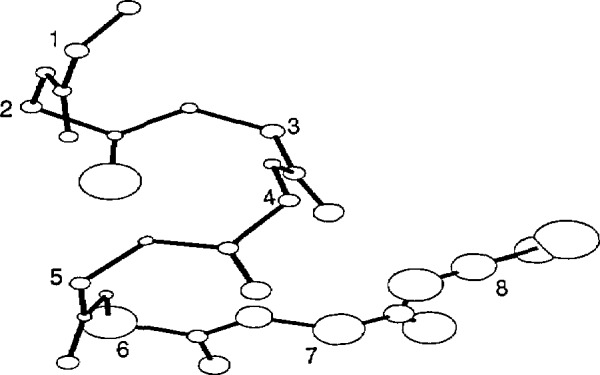
Helix-turn 8 residue stage H template. Sphere size represents 50% of the rmsd error at each atomic position. The Ca atom of each residue is numbered. Picture generated by the PLUTO program.

**Figure 3:**
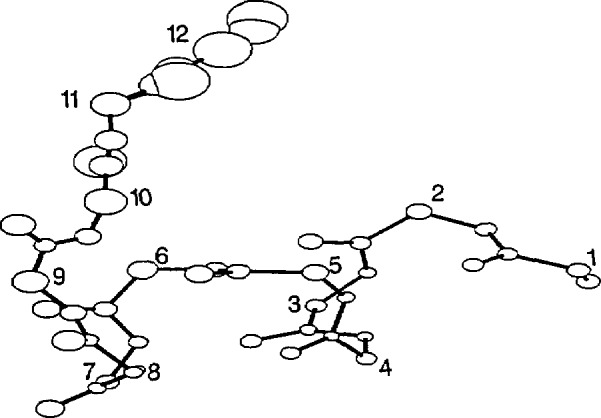
Helix-turn 12 residue stage II template. Sphere size represents 50% of the rmsd error at each atomic position.

**Figure 4:**
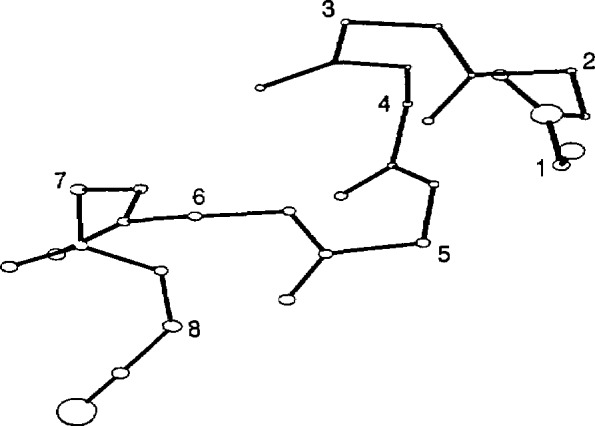
Turn-helix 8 residue stage II template. Sphere size represents 50% of the rmsd error at each atomic position.

**Figure 5:**
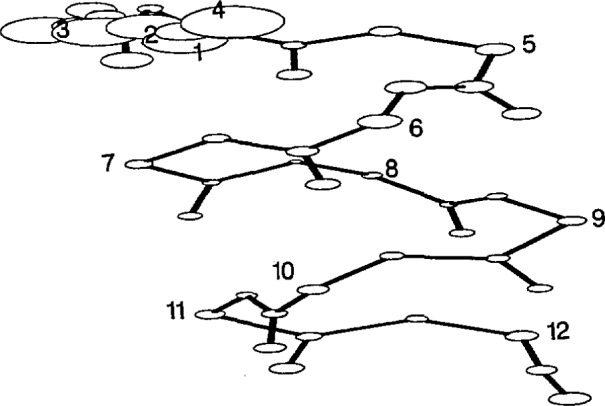
Turn-helix 12 residue stage II template. Sphere size represents 50% of the rmsd error at each atomic position.

The average structure of the HT8 stage II template is shown in figure 2, HT12 in figure 3, TH8 in figure 4 and TH12 in figure 5. The sphere centered at each atom represents 50% of the standard deviation error in atomic position at that atom between all members used to generate the stage II template. The templates were relatively structurally homologous. The helix atoms in both 8 residue templates had an error (0.30 ±0.1 Å) close to the experimental error of the protein coordinate sets whereas the atoms associated with the turn were less well defined (0.4±0.2 Å). The longer 12 residue templates were less accurate with an average error of 0.7 ±0.3 Å in the turn regions, double that of the helix region (0.3±0.1 Å). The associated *X, Y, Z* coordinates are given in Appendix 1: phi, psi backbone angles of each template are given in table 3. Residues in the turn did not correspond to any of the standard *β* turn types.

The sequences of the top 100 residues used to generate the stage II template were compiled and subjected to PROFILE analysis. The profiles are given in Appendix 2, consensus sequences are shown in table 4. Standard weighting, a gap penalty of 3.0 and a length penalty of 0.1 was used throughout. The sequences of 64 non-homologous structures were used to generate the helix-turn 8 profile, 51 for HT12, 36 for TH8 and 35 for TH12.

The PIR database of amino acid sequences was scanned for sequences which had a close alignment to that of each sequence profile. The alignment of the profile to an amino acid sequence was scored on the basis of the Dayhoff evolutionary metric matrix with a penalty factor for each gap [37].

One restriction of the PROFILESEGMENT program, as currently implemented, is that only the “best” alignment found for each protein is reported. Consequently, the procedure does not report multiple occurrences of a close alignment to the profile in one protein. Table 5 shows the alignment scores of each profile to the database. The score for TH12 was significantly better for the best 100 hits to the PIR database versus the entire database. This was due to a single segment of hemoglobin as identified by the TH12 profile. Since there are more than 100 variants of hemoglobin in the PIR database this search score was artificially high.

The ability of the profiles to correctly identify structural elements in amino acid sequences is summarized in table 6. The 12 residue templates had, on average, a higher discriminatory power than the 8 residue templates. In neither case were the profiles useful for predictive purposes. The number of sequences which were incorrectly identified as the “best” hit by PROFILEGAP was high at some 50%. Since only one hit is reported it is uncertain if any of the segments classified under “Multiple” in table 6 could be correctly identified by this procedure.

## 4. Discussion

The ability of a given protein sequence to rapidly and reproducibly adopt a single major backbone fold is believed to be inherent to its linear amino acid code. However, the initial sequence-specific signals which are associated with the initiation of the folding process are still unknown. Routes or pathways of folding have been proposed for a number of proteins [13]. Certain sites (e.g., certain turns stabilized by a few hydrogen bonds) have a higher degree of structural compactness and may be the primary cores at which folding was originated. The events associated with subsequent side-chain/side-chain stabilizations and further main-chain hydrogen bonds are only open to speculation at this point.

To make the transition between a novel linear amino acid sequence and a three-dimensional structure the protein modeler will need to be able to identify the critical sites necessary for the determination of the overall fold of the protein. This requires, however, the availability of coordinate sets for compact structures and the range of amino acids which can be used to create these sequences.

It is difficult, at this time, to assign structural elements from a protein to an average coordinate template from a family of possibilities. In this work, a rather arbitrary cutoff of a high rmsd of main-chain atoms was chosen. This may not be a very sensitive indicator of structural homology. Application of cluster analysis to side-chain atom contact plots, or to side-chain rmsd values, along with solvent accessibility values at each residue may be useful to help further categorize the fragments and thus better define the template [38], The accuracy of the turn-helix 8 template in the turn region as compared to the relative diffuseness at the turn region of the turn-helix 12 template illustrates this point well. Also, template definition may be improved during the superimposition procedure. In this work a rigid body rotation/translation algorithm was applied. An alternative would be to use a dynamic algorithm which could allow for breaks in the backbone chain during superimposition [39]. This will be of particular importance for the preparation of larger domain templates.

Once a particular structural template has been defined all sequences which give rise to it can be readily identified. The variability of the amino acids at each residue position over the template region is known as its sequence profile. These profiles are dependent upon the correct sequence alignment among many proteins. Obviously, knowledge of the structure is the ultimate check of the sequence alignment. Application of the standard Needleman-Wunsch algorithm to a small number of sequences will continue to suffer from the well-known alignment problem in which residues that occupy the same three-dimensional volume are often not equated. As a rule of thumb, if the structure is unknown but some 20+ homologous sequences are known, the correct alignment can probably be achieved.

In the absence of structure, a diagnostic sequence profile can still be prepared for certain elements. For example, the consensus profile for the DNA binding zinc finger motif has been defined [13,40].

The metric matrix of Dayhoff (based upon evolutionary relationships) which is used during the sequence alignment procedure may not be appropriate in all cases. It has been shown, in certain structural elements, that otherwise conservative replacements are not possible. For example, the replacement of aspartic acid by glutamic acid is not possible at the N-cap position of an *α* helix [16].

The identification, preparation, and application of these profiles is still a matter of some debate [41]. For example, if the domain of interest is large, as in the case of a globin fold, it is a reasonably straight-forward matter to achieve a correct sequence alignment among many homologous sequences. To be useful for the modeling of proteins *de novo*, significantly shorter domains or substructural elements must be accurately identified: the profile sequences of elements such as *α* helices or *β* turns may not be sufficiently specific to discriminate their existence in a sequence. The procedure may thus be limited to finding only a few very specific substructural elements or large folded domains.

If a specific element or fold has been identified from a given structure, a statistically large sample of sequences relating to the template will be required to show the range of residues which can occupy any particular site. The databases of structure and sequences may still be too small to allow for statistical certainty at this time [41].

In the next stage of model building the zones of known structure are joined together to create a range of folding possibilities [42,43]. All the residues are set to alanine except for glycine and proline: this restricts the number of degrees of freedom in the folding problem. Distance geometry or combinatorial approaches can be used to fold the backbone [44]. This is a severely underdetermined system and additional information is certainly needed to constrain the system. The principal restrictions used to restrain the system can be understood easily enough: no atomic overlap; residues should be closely packed; hydrogen bonds are often formed [45]; charged residues are most often found on the surface [46]; restricted conformational possibilities for disulfide bonds [47] and proline residues [48]; sequence dependant statistical data [49,50] such as (flexibility, hydrophilicity, surface accessibility); side-chain volumes; average number of contacts for residues in given substructural regions [36]; Ramachandran plot preferences for phi, psi angles; and any known biochemical information such as disulfide bonding patterns, or specific residues which come together to form an active site.

A major assumption of this approach is that interactions between defined sub-structural domains will affect primarily the details of the side-chain packings [51]: the backbone configuration will remain relatively constant during subsequent model building steps. The placement of side-chains *de novo* is clearly a very difficult job. However, various models have hand-built the core of a protein with surprising ease [52,53]. The methodology to discriminate between competing core packing motifs is still under development. This level of precision, in the preparation of models, is beyond the scope of this work.

These models will be of interest from a variety of standpoints. First, by comparing the variety of ways of joining structural fragments it may be possible to identify why certain motifs are favoured in nature. That is, certain amino acids at specific points may lead to one particular fold. This can be seen most clearly with the role of glycine in allowing certain turn types to exist. Also, the refinement of x-ray crystal structures can also benefit from this approach. A current version of the graphics program FRODO incorporates a library of fragments which can be laid into the electron density map and thus help speed the process of interpretation and refinement [54].

A library of average secondary and super-secondary templates and their associated sequence profiles is currently in preparation. Due to the small size of the databases, the discriminatory power of these profiles may be low. However, the average coordinate sets will still be very useful for general modeling purposes.

## Figures and Tables

**Table 1:** Residues used in the generation of stage 1 templates

Helix-turn 8		Helix-turn 12		Turn-helix 8		Turn-helix	12
Range	File^a^	Range	File	Range	File	Range	File
6–13	2ACT	35–46	2ACT	98–105	2ACT	19–30	2ACT
75–82	2ACT	122–133	2ACT	13–20	5CPA	89–100	5CPA
116–123	5CPA	227–238	5CPA	95–102	4DFR	97–108	3CPV
242–249	5CPA	255–266	5CPA	38–45	3FXN	90–101	3CYT
28–36	3CPV	99–110	3FXN	92–99	3FXN	105–116	6LYZ
9–16	3CYT	142–153	3MBN	3–10	6LYZ	45–56	4PTI
31–38	6LYZ	35–46	8PAP	78–85	6LYZ	2–13	5RSA
92–99	3MBN	119–130	8PAP	2–9	3MBN	297–308	3TLN
6–13	8PAP	98–109	2SNS	99–106	3MBN		
73–80	8PAP			123–130	3MBN		
14–21	ISBT			1–8	4PTI		
147–154	3TLN						
240–247	3TLN						
268–275	3TLN						
Average rmsd of superimposed main-chain atomic coordinates (Å)							
1.79 ±0.54^b^		2.67±1.15		1.08±0.40		2.80±0.86	
Average rmsd of superimposed side-chain atomic coordinates (Å)^c^							
2.13+0.83		3.85+1.61		1.72+0.65		3.92±1.13	
Average number of side-chain atom superimposed over the entire template							
12.0±3.8		16.8 + 3.6		12.9+4.0		17.3±6.7	

a Brookhaven code.

b Error expressed as standard deviation.

c Side-chain coordinates were checlced between superimposed structures if their atomic name was the same.

**Table 2:** rmsd of fragments extracted from the Brookhaven database to stage I coordinates

Template	Top 50 fragments		Top 100 fragments	
	rmsd (Å)	*±*^a^ (Å)	rmsd (Å)	± (Å)
Helix-turn 8	0.85	0.04	0.92	0.08
Helix-turn 12	1.45	0.09	1.54	0.12
Turn-helix 8	0.38	0.02	0.41	0.03
Turn-helix 12	1.36	0.03	1.40	0.05

a Error expressed as standard deviation.

**Table 3:** Backbone phi, psi angles of the stage II templates

	Helix-turn 8		Helix-turn 12		Turn-helix 8		Turn-helix	12
Residue no.s	Phi	Psi	Phi	Psi	Phi	Psi	Phi	Psi
1		−41.0		−41.9		144.0		40.9
2	−65.5	−41.7	−61.9	−40.1	−72.7	151.2	−111.6	16.5
3	−65.0	−37.0	−62.3	−40.6	−55.4	−37.6	−87.5	−146.1
4	−71.6	−43.5	−63.7	−43.2	−62.1	−39.8	−58.6	−444
5	−74.7	−35.4	−62.8	−40.1	−69.1	−37.0	−64.6	−43.0
6	−99.3	−15.3	−63.6	−38.6	−66.5	−39.6	−66.7	−39.5
7	93.3	53.4	−65.2	−30.8	−66.5	−35.1	−60.1	−43.7
8			−88.7	−15.1			−62.5	−41.8
9			107.2	23.5			−646	−44.0
10			−106.4	164.6			−647	−40.7
11			−84.5	149.8			−62.0	−41.2
12								

**Table 4:** Consensus sequence of each profile with most likely amino acids at each residue position^a^

	Residue number											
	1	2	3	4	5	6	7	8	9	10	11	12
HT8	hpl^b^	L	m,l	k	hpl	k	G	m				
HT12	e	A	a	hpb^c^	L	k,q	hpl	hpb	G	.^d^	x^e^	V
TH8	L	S	e,d	S,G	B,D,N	y	K	S				
TH12	hpl	.	T	A	E,D	V	a	A	A	L.M	k,q	K

a A capital letter (one letter amino acid code) signifies a weighting factor of ⩾ 0.5; lowercase is weighting ⩾ 0.3 and < 0.5.

b hpl—hydrophilic amino acids.

c hpb—hydrophobic amino acids.

d —no amino acids had a weighting factor ⩾ 0.3.

e The amino acid set a, b, d, e, t g, k, p, s, t all had a 0.3 weighting.

**Table 5:** Profile search of amino acid sequence databases

Template	Maximum score^a^	Protein Identification Resource Database		
		All entries^b^	Top 100^c^	Brookhaven database^d^
Helix-turn B	3.30	2.31±0.30	2.87±0.08	2.33+0.28
Helix-turn 12	5.10	3.26+0.44	4.02+0.59	3.36±0.35
Turn-helix 8	4.70	3.04±0.42	3.78±0.70	3.10+0.37
Turn-helix 12	6.20	3.84±0.62	5.54±0.07	402+0.63

a Score is based upon alignment metric matrix of the number of conserved residues less a penalty for introduced gaps.

b Average score of all 6862 sequences in release 15.0 of the PIR database.

c Average score for the 100 sequences which matched closest to the profile.

d Average score for the 82 sequences which are the non-homologous sequences corresponding to known structures in the Brookhaven database of better than 2.5 Å resolution.

**Table 6:** Distribution of the “best” hits found by each profile sequence^a^

Number of sequences found				
	Helix-turn 8	Helix-turn 12	Turn-helix 8	Turn-helix 12
Found	5 (7.8%)	6 (11.7%)	4 (11.1%)	6 (17.1%)
Missed	32 (50.0%)	21 (41.2%)	16 (44.4%)	18 (51.4%)
Multiple^b^	27 (42.2%)	24 (47.1%)	16 (44.4%)	11 (44.4%)

a Checked against a database of 82 unique sequences which relate to the non-homologous entries in the Brookhaven database of resolution <2.5 Å.

b If multiple entries of a structural element exist within a protein only the best hit is reported by PROFILEGAP. The number of extra entries which could not be found are listed as “Multiple”.

## Appendix 1. Stage II Template Coordinates with an Average Standard Deviation Derived from the Coordinates Used to Create the Template

**Table:** Helix-turn 8

Atom		Residue				
No.	Type	No.	*X*	*Y*	*Z*	Std dev
1	N	1	−0.231	−1.983	6.100	0.3536
2	CA	1	−1.279	−2.473	5.293	0.3663
3	C	1	−1.670	−1.514	4.204	0.2730
4	O	1	−1.878	−1.884	3.090	0.2863
5	N	2	−1.715	−0.263	4.546	0.2890
6	CA	2	−2.045	0.777	3.600	0.3193
7	C	2	−0.999	0.886	2.546	0.2400
8	O	2	−1.331	1.030	1.389	0.9320
9	N	3	0.229	0.799	2.934	0.2600
10	CA	3	1.304	0.896	1.998	0.3640
11	C	3	1.288	−0.262	1.026	0.3410
12	O	3	1.570	−0.097	−0.127	0.4650
13	N	4	0.939	−1.4fl8	1.507	0.2603
14	CA	4	0.883	−2.585	0.691	0.3250
15	C	4	−0.287	−2.530	−0.268	0.3126
16	O	4	−0.161	−2.870	−1.405	0.4597
17	N	5	−1.399	−2.123	0.189	0.2237
18	CA	5	−2.603	−2.098	−0.605	0.3057
19	C	5	−2.655	−1.002	−1.620	0.2207
20	O	5	−3.177	−1.174	−2.674	0.3367
21	N	6	−2.130	0.097	−1.302	0.2103
22	CA	6	−2.170	1.238	−2.173	0.8410
23	C	6	−0.962	1.446	−2.931	0.3200
24	O	6	−0.842	2.099	−3.812	0.4777
25	N	7	−0.067	0.907	−2.602	0.5613
26	CA	7	1.102	1.030	−3.268	0.7877
27	C	7	2.119	1.717	−3.210	0.4600
28	O	7	2.510	2.168	−3.743	0.8210
29	N	8	2.535	1.783	−2.557	0.8350
30	CA	8	3.534	2.396	−2.419	0.6900
31	C	8	4.547	2.529	−2.273	0.6777
32	O	8	5.041	2.469	−2.123	0.9867

**Table:** Turn-helix 8

Atom		Residue				
No.	Type	No.	*X*	*Y*	*Z*	Std dev
1	N	1	3.667	0.616	6.610	0.3767
2	CA	1	3.517	0.284	5.297	0.2450
3	C	1	3.322	1.484	4.418	0.4580
4	O	1	2.666	2.419	4.814	0.2460
5	N	2	3.860	1.427	3.246	0.1547
6	CA	2	3.676	2.481	2.262	0.1453
7	C	2	2.261	2.433	1.709	0.1257
8	O	2	1.623	1.370	1.672	0.1637
9	N	3	1.771	3.575	1.305	0.1207
10	CA	3	0.443	3.688	0.710	0.1360
11	C	3	0.281	2.769	−0.484	0.1067
12	O	3	−0.790	2.179	−0.670	0.1417
13	N	4	1.330	2.632	−1.261	0.1073
14	CA	4	1.327	1.777	−2.427	0.1503
15	C	4	1.094	0.326	−2.074	0.1470
16	O	4	0.347	−0.367	−2.754	0.1967
17	N	5	1.687	−0.119	−0.996	0.1573
18	CA	5	1.523	−1.484	−0.538	0.2087
19	C	5	0.120	−1.715	−0.035	0.2083
20	O	5	−0.442	−2.786	−0.229	0.2430
21	N	6	−0.406	−0.711	0.601	0.1953
22	CA	6	−1.758	−0.803	1.105	0.2440
23	C	6	−2.778	−0.889	−0.008	0.2023
24	O	6	−3.750	−1.648	0.070	0.2610
25	N	7	−2.539	−0.133	−1.032	0.2057
26	CA	7	−3.424	−0.139	−2.184	0.2483
27	C	7	−3.393	−1.456	−2.907	0.2007
28	O	7	−4.418	−1.928	−3.388	0.2500
29	N	8	−2.253	−2.063	−2.938	0.2070
30	CA	8	−2.109	−3.359	−3.553	0.2777
31	C	8	−2.861	−4.421	−2.817	0.2540
32	O	8	−3.486	−5.278	−3.413	0.3157

**Table:** Helix-turn 12

Atom		Residue				
No.	Type	No.	*X*	*Y*	*Z*	Std dev
1	N	1	6.111	3.843	−3.190	0.4603
2	CA	1	6.583	2.570	−2.619	0.4447
3	C	1	5.401	2.531	−1.748	0.3917
4	o	1	4.618	1.588	−1.770	0.4111
5	N	2	5.256	3.543	−1.006	0.4167
6	CA	2	4.159	3.630	−0.128	0.4980
7	C	2	2.846	3.630	−0.836	0.4110
8	O	2	1.891	2.995	−0.433	0.4817
9	N	3	2.810	4.300	−1.894	0.3430
10	CA	3	1.619	4.364	−2.674	0.4053
11	C	3	1.201	3.041	−3.200	0.3360
12	O	3	0.036	2.665	−3.225	0.4160
13	N	4	2.151	2.335	−3.606	0.3030
14	CA	4	1.915	1.028	−4.114	0.3960
15	C	4	1.370	0.129	−3.092	0.3000
16	O	4	0.436	−0.637	−3.316	0.3800
17	N	5	1.937	0.232	−1.976	0.3350
18	CA	5	1.494	−0.577	−0.918	0.5107
19	C	5	0.098	−0.310	−0.534	0.4610
20	O	5	−0.706	−1.198	−0.297	0.5497
21	N	6	−0.211	0.905	−0.536	0.4593
22	CA	6	−1.528	1.305	−0.216	0.5830
23	C	6	−2.545	0.807	−1.186	0.4630
24	O	6	−3.641	0.399	−0.837	0.5450
25	N	7	−2.180	0.818	−2.381	0.3763
26	CA	7	−3.062	0.384	−3.413	0.4400
27	C	7	−3.411	−1.052	−3.342	0.3443
28	O	7	−4.461	−1.475	−3.681	0.5333
29	N	8	−2.561	−1.782	−2.878	0.2570
30	CA	8	−2.779	−3.177	−2.729	0.3543
31	C	8	−3.386	−3.530	−1.452	0.3693
32	O	8	−3.820	−4.442	−1.241	0.6383
33	N	9	−3.419	−2.846	−0.625	0.5010
34	CA	9	−3.988	−3.092	0.602	0.6870
35	C	9	−3.472	−3.302	1.694	0.4170
36	O	9	−3.852	−3.763	2.527	0.6613
37	N	10	−2.597	−2.934	1.708	0.5007
38	CA	10	−2.020	−3.047	2.731	0.8130
39	C	10	−1.676	−2.232	3.735	0.6440
40	O	10	−1.700	−1.500	3.789	1.0103
41	N	11	−1.407	−2.366	4.529	0.6257
42	CA	11	−1.084	−1.632	5.543	0.7487
43	C	11	−0.016	−1.078	5.836	0.7463
44	O	11	0.371	−1.087	5.826	1.1897
45	N	12	0.449	−0.618	6.098	0.8347
46	CA	12	1.486	−0.070	6.444	1.1203
47	C	12	2.243	0.245	6.887	0.9610
48	O	12	2.383	0.463	7.146	1.1600

**Table:** Turn-helix 12

Atom		Residue				
No.	Type	No.	*X*	*Y*	*Z*	Std dev
1	N	1	−0.735	5.059	7.604	1.1313
2	CA	1	−0.748	4.906	7.024	0.8767
3	C	1	−1.257	4.780	5.974	0.6573
4	o	1	−1.255	4.475	5.638	0.9323
5	N	2	−1.683	5.016	5.458	0.8573
6	CA	2	−2.198	4.889	4.410	1.003
7	C	2	−2.401	5.125	3.289	0.5843
8	O	2	−2.793	5.004	2.870	0.9253
9	N	3	−2.111	5.432	2.839	0.4377
10	CA	3	−2.258	5.666	1.791	0.549
11	C	3	−1.915	4.971	0.577	0.3917
12	O	3	−1.901	3.838	0.498	0.573
13	N	4	−1.619	5.645	−0.365	0.2827
14	CA	4	−1.259	5.093	−1.611	0.2787
15	C	4	−0.082	4.174	−1.563	0.2807
16	O	4	−0.090	3.125	−2.116	0.3837
17	N	5	0.916	4.537	−0.889	0.358
18	CA	5	2.096	3.735	−0.759	0.4317
19	C	5	1.864	2.431	−0.036	0.434
20	O	5	2.366	1.388	−0.428	0.4293
21	N	6	1.127	2.492	1.011	0.4647
22	CA	6	0.826	1.310	1.771	0.492
23	C	6	−0.029	0.358	0.992	0.3647
24	O	6	0.181	−0.856	1.042	0.391
25	N	7	−0.960	0.905	0.272	0.3097
26	CA	7	−1.816	0.097	−0.542	0.309
27	C	7	−1.036	−0.670	−1.554	0.2067
28	O	7	−1.271	−1.844	−1.782	0.2703
29	N	8	−0.108	0.001	−2.161	0.1563
30	CA	8	0.720	−0.614	−3.156	0.232
31	C	8	1.547	−1.747	−2.587	0.2053
32	O	8	1.678	−2.792	−3.187	0.2853
33	N	9	2.069	−1.532	−1.443	0.2403
34	CA	9	2.873	−2.525	−0.774	0.324
35	C	9	2.061	−3.765	−0.411	0.27
36	O	9	2.502	−4.896	−0.620	0.2783
37	N	10	0.915	−3.533	0.088	0.278
38	CA	10	0.039	−4.627	0.457	0.3543
39	C	10	−0.393	−5.429	−0.719	0.2913
40	O	10	−0.458	−6.652	−0.669	0.3537
41	N	11	−0.686	−4.748	−1.776	0.2387
42	CA	11	−1.093	−5.406	−2.971	0.3363
43	C	11	−0.027	−6.302	−3.511	0.2957
44	O	11	−0.287	−7.416	−3.939	0.3897
45	N	12	1.158	−5.818	−3.454	0.2563
46	CA	12	2.280	−6.571	−3.900	0.368
47	C	12	2.481	−7.825	−3.088	0.341
48	O	12	2.767	−8.885	−3.593	0.462

## Appendix 2. Sequence Profiles for Each Template

**Table:** Helix-turn 8

Amino acid																								

Residue		A	B	C	D	E	F	G	H	I	K	L	M	N	P	Q	R	S	T	V	W	X	Y	Z
No.	Type^a^																							
1	E	0.4	0.3	−0.1	0.4	0.4	−0.3	0.3	0.1	0.2	0.1	0.1	0.1	0.2	0.1	0.4	0.0	0.2	0.1	0.2	−05	0.1	−0.2	0.4
2	L	0.2	−0.1	−0.2	−0.1	−0.1	0.4	0.0	−0.1	0.4	−0.1	0.6	0.5	−0.1	−0.1	0.0	−0.1	0.1	0.1	0.4	0.0	0.1	0.1	−0.1
3	L	0.1	0.1	−0.2	0.0	0.1	0.1	0.0	0.2	0.2	0.0	0.3	0.3	0.1	0.0	0.2	0.1	0.0	0.1	0.2	−0.1	0.1	0.0	0.1
4	K	0.2	0.1	−0.1	0.1	0.2	−0.1	0.1	0.1	0.2	0.3	0.1	0.2	0.1	0.1	0.1	0.1	0.1	0.2	0.2	−0.2	0.1	−0.1	0.2
5	E	0.3	0.3	0.0	0.3	0.3	−0.2	0.2	0.2	0.0	0.2	−0.1	0.0	0.3	0.1	0.2	0.1	0.3	0.2	0.0	−0.2	0.1	−0.1	0.3
6	K	0.2	0.2	−0.1	0.1	0.1	−0.1	0.1	0.1	0.1	0.3	0.1	0.2	0.2	0.1	0.2	0.1	0.2	0.1	0.1	0.0	0.1	−0.1	0.1
7	G	0.4	0.5	−0.1	0.5	0.4	−0.3	0.8	0.1	−0.1	0.0	−0.2	0.0	0.4	0.2	0.3	−0.1	0.3	0.2	0.2	−0.6	0.1	−0.4	0.3
8	M	0.1	0.1	−0.2	0.0	0.0	0.1	0.0	0.1	0.2	0.2	0.2	0.3	0.1	0.0	0.1	0.1	0.1	0.1	0.2	0.1	0.1	0.0	0.1
Total^b^		72	0	14	35	34	29	74	43	32	68	87	31	31	4	41	30	50	24	42	6	10	27	0

a This amino acid was identified as the consensus amino acid by profile.

b Total number of each amino acid used in the generation of the profile.

**Table:** Helix-turn 12

Amino acid																								

Residue		A	B	C	D	E	F	G	H	I	K	L	M	N	P	Q	R	S	T	V	W	X	Y	Z
No.	Type^a^																							
1	E	0.3	0.2	0.1	0.3	0.4	−0.1	0.3	0.0	0.2	0.1	0.0	0.0	0.2	0.1	0.1	−0.1	0.3	0.3	0.2	−05	0.1	−0.2	0.2
2	A	0.5	0.2	0.1	0.2	0.3	−0.2	0.3	0.1	0.2	0.1	0.1	01	0.2	0.2	0.2	−0.1	0.2	0.3	0.2	−04	0.1	−0.2	0.2
3	A	0.4	0.2	−0.2	0.3	0.3	−0.2	0.2	0.2	0.0	0.1	0.1	01	0.3	0.1	0.3	0.1	0.2	0.1	0.1	−02	01	−0.1	0.3
4	L	0.1	−0.1	−0.1	−0.1	−0.1	0.3	−0.1	0.0	0.4	0.0	0.4	04	0.0	−0.1	−0.1	−0.1	0.0	0.1	0.4	0.0	01	0.2	−0.1
5	L	0.2	−0.1	−0.1	−0.1	0.0	0.3	−0.1	0.0	0.3	0.0	0.5	04	0.0	−0.1	0.0	−0.1	0.0	0.2	0.3	−0.1	01	0.1	0.0
6	K	0.1	0.2	−0.4	0.2	0.2	−0.1	0.0	0.2	0.1	0.4	0.2	0.3	0.2	0.0	0.4	0.2	0.0	0.1	0.1	−0.1	01	−0.2	0.3
7	E	0.4	0.4	0.0	0.4	0.4	−0.2	0.3	0.1	0.0	0.1	0.0	0.0	0.4	0.1	0.2	−0.1	0.2	0.2	0.0	−0.4	01	−0.1	0.3
8	V	0.3	0.0	0.1	0.0	0.0	0.2	0.1	0.0	0.3	0.0	0.3	0.3	0.0	0.1	0.0	−0.1	0.2	0.2	0.3	0.0	01	0.0	0.0
9	G	0.4	0.5	0.0	0.6	0.4	−0.5	0.8	0.1	−0.2	0.1	−0.3	−0.1	0.4	0.3	0.4	−01	0.3	0.4	0.1	−0.7	0.1	−0.5	0.4
10	A	0.2	0.1	0.2	0.1	0.0	01	0.2	0.0	01	0.0	0.1	0.1	0.1	0.0	0.0	−01	0.2	0.1	0.1	0.0	0.1	0.1	0.0
11	T	0.3	0.3	0.0	0.3	0.3	−0.4	0.3	0.1	0.1	0.3	−0.1	0.0	0.2	0.3	0.2	0.1	0.3	0.3	0.2	−0.4	0.1	−0.3	0.2
12	V	0.3	0.2	0.0	0.2	0.2	−0.1	0.3	0.0	0.3	0.0	0.2	0.2	0.1	0.2	0.1	−0.1	0.2	0.2	0.5	−0.5	0.1	−03	0.2
Total^b^		120	0	11	35	74	22	103	17	59	64	98	30	60	25	69	30	70	65	88	12	15	72	1

a This amino acid was identified as the consensus amino acid by profile.

b Total number of each amino acid used in the generation of the profile.

**Table:** Turn-helix 8

Amino acid																								

Residue		A	B	C	D	E	F	G	H	I	K	L	M	N	P	Q	R	S	T	V	W	X	Y	Z
No.	Type^b^																							
1	L	0.0	−0.2	−0.2	−0.2	0.0	0.6	−0.2	0.0	0.5	−0.1	0.7	0.6	−0.1	−0.1	0.0	−0.2	−0.1	0.0	0.5	0.0	0.1	0.2	0.0
2	S	0.4	0.3	0.4	0.2	02	−0.3	0.5	−0.1	0.0	0.2	−0.3	−0.2	0.3	0.4	0.0	0.1	1.0	0.5	0.0	−0.1	0.1	−0.4	0.1
3	D	0.3	03	−0.2	0.4	0.4	−0.2	0.2	0.2	0.1	0.1	0.1	01	0.3	0.2	0.3	0.0	0.2	0.2	0.1	−0.4	0.1	−0.2	0.3
4	G	0.5	0.4	0.0	0.5	0.5	−0.5	0.6	0.0	−0.1	0.3	−0.3	−0.1	0.3	0.3	0.2	0.0	0.6	0.4	0.0	−0.5	0.1	−0.4	0.4
5	N	0.3	0.6	0.0	0.6	0.5	−03	0.4	0.3	−01	0.2	−0.2	−0.2	0.6	0.1	0.3	0.0	0.4	0.2	−0.1	−0.3	0.1	−0.2	0.4
6	Y	0.0	−0.3	0.0	−0.4	−0.4	0.4	−0.1	−0.1	0.3	−0.1	0.3	0.1	−0.2	−0.1	−0.2	0.1	0.2	0.0	0.3	−0.1	0.0	0.4	−0.3
7	K	0.2	0.2	−0.3	0.2	0.3	−0.2	0.1	0.1	0.1	0.5	0.1	0.2	02	0.0	0.3	0.2	0.1	0.2	0.1	−02	0.1	−02	0.3
8	S	0.2	0.1	0.1	0.1	0.1	−0.1	0.2	0.0	0.0	0.2	0.0	0.0	0.2	0.1	0.0	0.1	0.5	0.2	0.0	−0.1	01	0.0	0.0
Total^b^		42	0	35	45	55	15	22	13	22	59	83	11	35	26	24	5	127	36	27	20	1	25	0

a This amino acid was identified as the consensus amino acid by profile.

b Total number of each amino acid used in the generation of the profile.

**Table:** Turn-helix 12

Amino acid																								

Residue		A	B	C	D	E	F	G	H	I	K	L	M	N	P	Q	R	S	T	V	W	X	Y	Z
No.	Type^b^																							
1	E	0.3	0.3	−0.1	0.3	0.3	−0.1	0.3	0.1	0.2	0.1	0.1	0.1	0.2	0.1	0.1	−0.1	0.2	0.2	0.2	−0.4	0.1	−0.1	0.2
2	Y	0.0	0.2	−0.3	0.1	0.1	0.2	0.2	0.1	0.1	−0.1	0.2	0.0	0.2	−0.1	0.0	−0.1	0.1	0.1	0.0	0.0	0.1	0.2	0.0
3	T	0.3	0.4	0.1	0.3	0.2	−0.3	0.5	0.0	0.1	0.2	−0.2	0.0	0.4	0.2	0.1	0.1	0.4	0.6	0.2	−0.4	0.1	−0.3	0.2
4	A	0.6	0.3	0.1	0.4	0.4	−0.5	0.5	0.1	−0.1	0.1	−0.3	−0.2	0.3	0.5	0.3	0.0	0.4	0.3	0.0	−0.8	0.1	−0.3	0.3
5	E	0.5	0.4	−0.2	0.6	0.6	−0.5	0.5	0.2	0.0	0.1	−0.1	−0.1	0.3	0.3	0.4	−0.1	0.2	0.2	0.1	−0.8	0.1	−0.3	0.5
6	V	0.2	0.0	−0.1	0.0	0.0	0.1	0.1	0.1	0.4	−0.1	0.4	0.4	0.0	0.1	0.1	−0.1	0.0	0.2	0.6	−0.3	0.1	0.0	0.0
7	A	0.4	0.2	−0.1	0.2	0.3	−0.1	0.2	0.2	0.1	0.1	0.1	0.2	0.2	0.2	0.3	0.0	0.2	0.2	0.2	−0.2	0.1	−0.2	0.3
8	A	0.8	0.3	0.2	0.3	0.2	−0.3	0.6	0.1	0.0	0.0	−0.1	0.0	0.3	0.3	0.1	−0.2	0.5	0.3	0.2	−0.4	0.1	−0.3	0.1
9	A	0.5	0.2	0.0	0.3	0.4	−0.2	0.3	0.1	0.2	0.1	0.0	0.1	0.2	0.2	0.2	−0.1	0.3	0.2	0.2	−0.5	0.1	−0.2	0.3
10	L	0.2	−0.1	−0.2	−0.2	−0.1	0.5	−0.2	−0.1	0.4	−0.1	0.6	0.6	−0.1	−0.2	−0.1	−0.1	−0.1	0.1	0.4	0.1	0.1	0.2	−0.1
11	K	0.1	0.3	−0.3	0.3	0.2	−0.2	0.1	0.2	0.1	0.4	0.1	0.2	0.3	0.1	0.4	0.3	0.1	0.1	0.1	−0.1	0.1	−0.3	0.3
12	K	0.2	0.3	−0.2	0.2	0.2	−0.4	0.2	0.1	0.0	0.6	−0.2	0.1	0.2	0.2	0.3	0.5	0.3	0.2	0.0	0.1	0.1	−0.5	0.3
Total^b^		168	0	15	61	98	54	90	33	20	61	91	22	67	39	40	52	67	60	95	1.4	3	26	0

a This amino acid was identified as the consensus amino acid by profile.

b Total number of each amino acid used in the generation of the profile.
